# In Silico and In Vitro Analysis of Major Cannabis-Derived Compounds as Fatty Acid Amide Hydrolase Inhibitors

**DOI:** 10.3390/molecules26010048

**Published:** 2020-12-24

**Authors:** Emanuele Criscuolo, Maria Laura De Sciscio, Filomena Fezza, Mauro Maccarrone

**Affiliations:** 1 Department of Experimental Medicine, Tor Vergata University of Rome, Via Montpellier 1, 00121 Rome, Italy; emanuele.criscuolo@alumni.uniroma2.eu; 2 Department of Medicine, Campus Bio-Medico University of Rome, Via Alvaro del Portillo 21, 00128 Rome, Italy; marialaura.desciscio@studio.unibo.it; 3 Department of Chemistry “G. Ciamician”, University of Bologna, Via Selmi 2, 40126 Bologna, Italy; 4 Department of Biotechnological and Applied Clinical Sciences, University of L’Aquila, Via Vetoio snc, 67100 L’Aquila, Italy; 5 European Center for Brain Research/IRCCS Santa Lucia Foundation, Via del Fosso di Fiorano 54, 00143 Rome, Italy

**Keywords:** cannabinoids, docking, endocannabinoids, FAAH, inhibition, modeling

## Abstract

Accumulated evidence suggests that enhancing the endocannabinoid (eCB) tone, in particular of anandamide (*N*-arachidonoylethanolamine, AEA), has therapeutic potential in many human diseases. Fatty acid amide hydrolase (FAAH) is a membrane-bound enzyme principally responsible for the degradation of AEA, and thus it represents a relevant target to increase signaling thereof. In recent years, different synthetic and natural compounds have been developed and tested on rat FAAH, but little is known of their effect on the human enzyme. Here, we sought to investigate six major cannabis-derived compounds to compare their action on rat and human FAAHs. To this aim, we combined an in silico analysis of their binding mode and affinity, with in vitro assays of their effect on enzyme activity. This integrated approach allowed to disclose differences in efficacy towards rat and human FAAHs, and to highlight the role of key residues involved in the inhibition of both enzymes. This study suggests that the therapeutic efficacy of compounds targeted towards FAAH should be always tested in vitro on both rat and human enzymes.

## 1. Introduction

**Figure 1:**
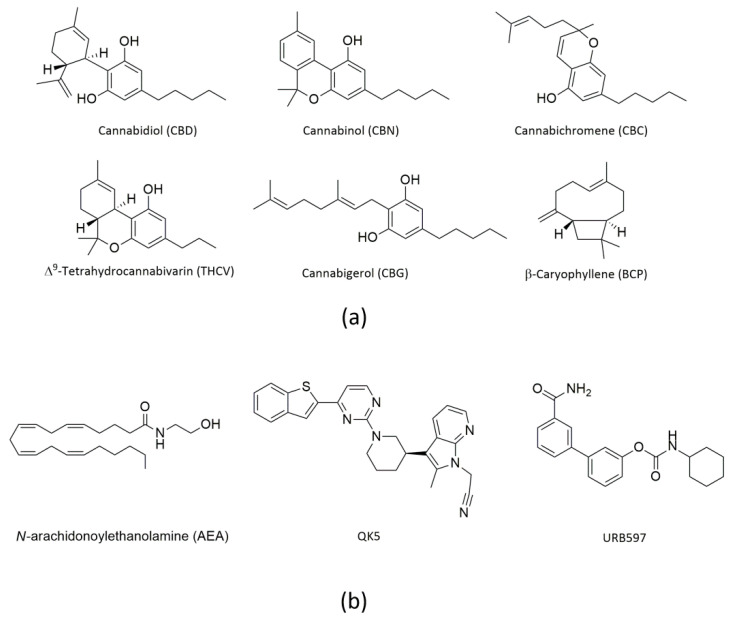
Chemical structures of (**a**) cannabis-derived compounds, and (**b**) AEA and two potent FAAH inhibitors, QK5 and URB597.

Cannabis (*Cannabis sativa* or *Cannabis indica*) is a fibrous plant of the Cannabaceae family, also known as hemp. It has been cultivated for a long time especially for textile use, but in recent years it has become much more popular as a recreational drug due to its psychotropic effects. Of note, cannabis extracts are used also as therapeutics to treat human diseases [1,2]. It should be noted that only ~120 of the >480 different substances present in cannabis are termed cannabinoids (or phytocannabinoids), which are all oxygen-containing C21 aromatic hydrocarbon compounds [3]. Of them, Δ^9^-tetrahydrocannabinol (THC) is the main psychoactive ingredient [2]. Additional cannabinoids with biological activity are cannabidiol (CBD), cannabinol (CBN), cannabichromene (CBC), Δ^9^-tetrahydrocannabivarin (THCV) and cannabigerol (CBG) [4,5], all shown in Figure 1a.

Indeed CBD, which represents ~40% of the plant resin extract, does not produce the typical psychotropic effects of THC [6], and has anti-inflammatory, anti-tumor, analgesic and anti-psychotic activity [7]. Remarkably, CBD has been approved for the treatment of seizures associated with Lennox–Gastaut syndrome, Dravet syndrome, or tuberous sclerosis complex in patients aged one year and older [8,9,10,11]. CBC shows antinociceptive and anti-inflammatory effects, and in vivo it potentiates some effects of THC [12]. CBG has antibacterial, antiproliferative, and bone-stimulant properties [13,14,15], whereas CBN is obtained by oxidation of THC in air and stimulates feeding in rats [16]. Finally, THCV has anorexic and antiepileptic effects, and it may be clinically effective to treat migraine [17].

Besides cannabinoids, terpenes and phenolic compounds are also present in cannabis extracts, and may bring health benefits. Among them, β-caryophyllene (BCP, shown in Figure 1a) has attracted attention as a selective, though unexpected, agonist of type 2 (CB_2_) but not type 1 (CB_1_) cannabinoid receptors [18]. BCP is a member of the sesquiterpene lactone family, and is found in large amounts in the essential oil of cannabis, as well as in curry, cloves, cinnamon and black pepper [19]. It has anti-diabetic, anti-inflammatory, hepatoprotective and neuroprotective properties in various experimental models [18,20].

**Figure 2:**
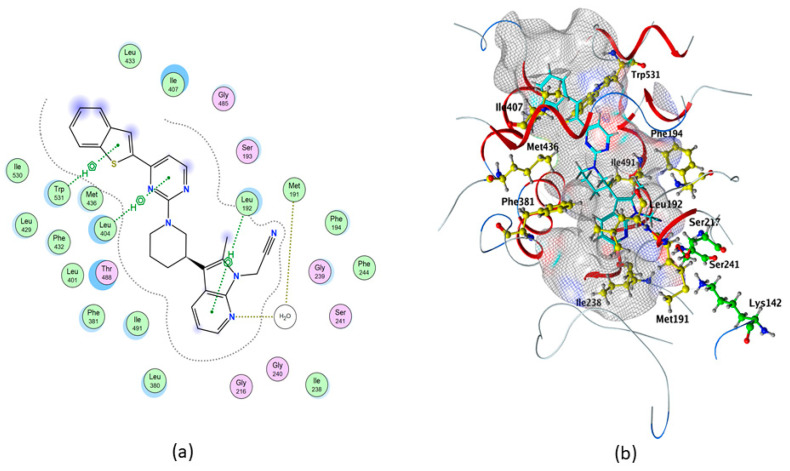
Best binding pose of QK5 within rFAAH, obtained through MOE analysis. (**a**) In the 2D structure the following details are shown: Pi–H interactions, in green; backbone acceptor, in blue; solvent contact, in yellow. (**b**) In the 3D structure the following elements are shown as sticks: QK5, in cyan; catalytic triad, in green; all residues involved in the protein/ligand interaction, in yellow.

It should be recalled that the biological activity of cannabis-derived compounds largely depends on their interaction with an ensemble of endogenous mediators, their receptors and metabolic enzymes, collectively termed endocannabinoid system (ECS) [21,22,23]. The latter is an evolutionarily conserved lipid signaling system, which comprises endocannabinoids (eCBs), such as anandamide (*N*-arachidonoylethanolamine, AEA) (Figure 1b) and 2-arachidonoylglycerol (2-AG), their target receptors (CB_1_ and CB_2_, but also transient receptor potential vanilloid 1, peroxisome proliferator-activated receptors α, δ and γ, and orphan G-protein-coupled receptors GPR119 and GPR55), and their metabolic enzymes and transport mechanisms [21,22,23]. In particular, the AEA-related ECS has major roles in human health and disease conditions, such as food intake, immune response, reproductive events, motor coordination and neurological disorders [2,22,24,25]. Remarkably, it is widely accepted that AEA signaling largely depends on the strict metabolic control of AEA concentration, especially played by the AEA-cleaving enzyme fatty acid amide hydrolase (FAAH) [24,26].

FAAH is a membrane-bound enzyme that was purified and cloned from rat liver membranes [27]. It is a homodimer with a highly conserved primary sequence that is rich in Ser and Gly residues and has a molecular mass of ~63 kDa per subunit. FAAH presents a Ser241-Ser217-Lys142 catalytic triad, and shows a strong preference for hydrophobic substrates [28,29,30]. Currently, various 3D structures of rat FAAH (rFAAH) have been reported and are largely used in biochemical and pharmacological studies [31]. These crystallographic structures reveal 11 twisted β-sheets in the center, surrounded by 24 α-helices of which α-18 and α-19 allow attachment of FAAH to the membrane [32]. Furthermore, the enzyme presents four cavities: (i) a membrane access (MA) channel, through which the substrates reach the catalytic site; (ii) an acyl-chain binding (ACB) cavity next to MA, which contributes to the correct orientation of the substrate during catalysis; (iii) an oxyanion hole (OH) close to catalytic triad, which stabilizes substrates in the correct orientation; and (iv) a cytosolic port (CP) associated with the catalytic region that allows the exit of the leaving group after substrate hydrolysis [33].

It is known that there are key residues for non-covalent rFAAH inhibition, located in the multiple binding cavities. In particular, in the MA there are two charged residues, Asp403 and Arg486, that together with Ile407 favor the entrance of the polar head group of the AEA substrate. Instead, the residues Leu192, Phe194, Phe381, Phe432, Met436 and Trp531 regulate the movement of the flexible arachidonoyl tail between MA and ACB; among them, Phe432 side chain rotation is a key switch, called “dynamic paddle” [26,32,34,35]. Furthermore, residues Ile238, Gly239, Gly240 and Ser241, located within OH, keep the substrate properly oriented for hydrolysis. Also Ile491 and Val495 appear to be essential for substrate binding [35,36], and Met191 seems to be central due to its ability to form a hydrogen bond between its CO group and the NH group of the substrate, allowing a nitrogen inversion essential for hydrolysis. Indeed, the presence of the polar residues (Ser218 and Thr236) favors the leaving group release, through a H-bond network with the protonated Lys142, after substrate hydrolysis [31,33,37,38]. Finally, Cys269 has a strategic position at the end of CP, where it maintains or enhances intrinsic inhibitor selectivity [39,40].

Unlike rFAAH, the 3D structure of human FAAH (hFAAH) has not yet been reported, due to solubility problems and tendency of this enzyme to aggregate [41,42]. However, the high homology (82%) between rFAAH and hFAAH allows, through homology modeling, the construction of a comparative model for the human enzyme [43]. In this context, it is important to stress that there are 6 different amino acids in rat versus human FAAH around the active site: Leu192, Phe194, Ala377, Ser435, Ile491, Val495 (in rFAAH), and Phe192, Tyr194, Thr377, Asn435, Val491 and Met495 (in hFAAH), respectively [44]. Thus, despite overt similarities in their sequences, these two enzymes seem to interact quite differently with exogenous molecules [41,42,45]. Unsurprisingly, several FAAH inhibitors that held promise in pre-clinical studies failed to become therapeutics for human disease [41].

To date, several studies have interrogated the effects of cannabis-derived compounds on the major eCB-binding receptors [4,5], whereas little is known on their potential interaction with rFAAH [46] or hFAAH [47]. Here, we sought to fill this gap by investigating the potential interaction of some relevant cannabis-derived compounds with both enzymes, through the combination of in silico computational analysis and in vitro activity assays. To this aim, we had to build up a molecular model of hFAAH that allowed us to compare in silico data and activity assays of this enzyme with those of rFAAH. Of note, docking analysis highlighted the ability of cannabis-derived substances to interact with both FAAHs in a non-covalent mode, yet with an apparent species-specific sensitivity.

## 2. Results

### 2.1. Molecular Docking of rFAAH

The structure of rFAAH deposited as 3QK5 [48,49] was retrieved from the Protein Data Bank (PDB) (www.rcsb.org). 3QK5 was co-crystallized with the potent non-covalent inhibitor QK5 (shown in Figure 1b), which yielded an half-maximal inhibitory concentration (IC50) value of 18 nM and was considered a control compound for in silico studies [48]. On this basis, we sought to validate the molecular docking algorithm by launching a re-docking analysis of QK5 into its original binding site, and obtained a superimposed pose with the co-crystallized control inhibitor (Figure S1). Then, we used the same protocol to dock all selected cannabis-derived compounds into the active site of rFAAH, and obtained the molecular data summarized in Table 1.

In particular, the control compound QK5 showed the highest binding affinity to rFAAH (ΔG = −10.337 kcal/mol), one acceptor water-mediated hydrogen bond (H-bond) with Met191, and three non-covalent interactions that involved π systems (pi–H) with Leu192, Leu404 and Trp531 (Table 1 and Table 2, and Figure 2), in keeping with previous findings [48].

In addition, we showed through the Molecular Operating Environment (MOE) software that QK5 makes hydrophobic interactions with Phe194, Ile238, Met436, Phe381, Ile407 and Ile491 (Table 2 and Figure 2).

Among cannabis-derived compounds, CBG showed the highest binding affinity (with ΔG = −8.9776 kcal/mol) without polar interactions (Table 1), and made three hydrophobic contacts with Trp531, Met436 and Ile407 (Table 2). CBD showed ΔG = −8.2035 kcal/mol, one water-mediated H-donor with Met191 (Table 1 and Table 2) and hydrophobic contacts with Phe381, Cys269, Leu192, Ile491 and Phe194 (Table 2). Also, CBC showed good affinity for rFAAH (ΔG = −7.9880 kcal/mol), one pi–H-bond with Leu380 (Table 1 and Table 2) and non-polar contacts with Leu192, Ile238, Phe432 and Ile491 (Table 2). CBN showed ΔG = −7.7719 kcal/mol, two polar interactions (i.e., pi–H with Phe381 and H-donor with Met436), and non-polar contacts with Trp531, Ile491, Met495 and Leu192 (Table 1 and Table 2). THCV showed ΔG = −7.6375 without polar interactions (Table 1), and made hydrophobic contacts with Leu192, Phe381, Phe432, Met436 and Ile491 (Table 2). Finally, BCP showed ΔG = −6.4016 kcal/mol (Table 1), and non-polar contacts with Leu192, Phe194, Ile238 and Ile491 (Table 2).

### 2.2. Homology Modeling of hFAAH

**Figure 3:**
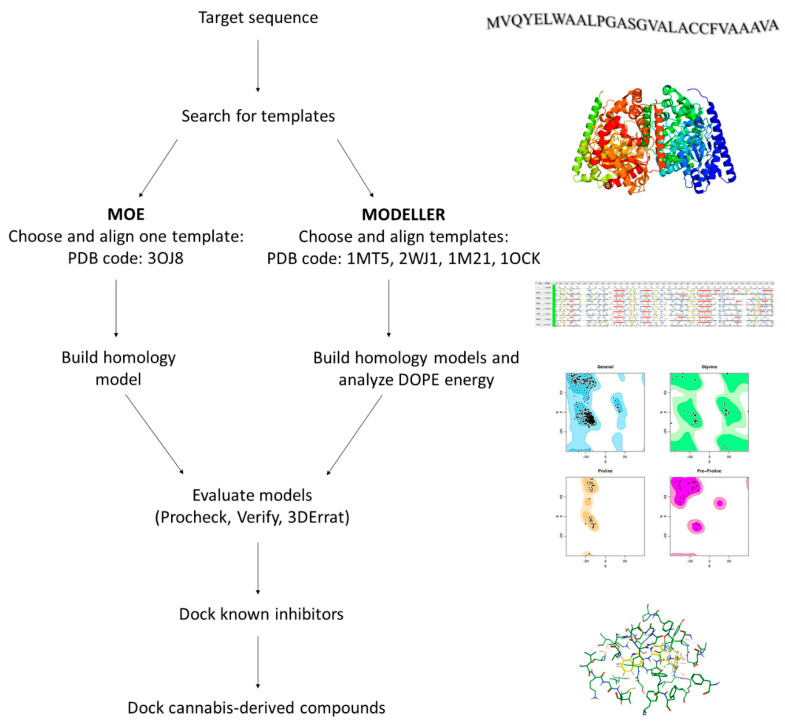
Flowchart of the homology modeling approach used to build the 3D structure of hFAAH.

To investigate the potential interactions of cannabis-derived compounds with hFAAH, we had to build and validate an unprecedented homology model of the enzyme; indeed, its 3D structure is not available in the PDB [49]. To generate hFAAH models we used both the MOE and MODELLER software, and the SWISS-MODEL and PHYRE2 web-servers [50,51,52,53], following the general computational strategy depicted in Figure 3.

A homology model is built starting from the target sequence as query, and then comparing it with a database; this analysis yields a list of potential templates, from which the structures with the highest sequence similarity can be chosen. Indeed, we chose from such a list the template with the highest identity and similarity (i.e., humanized-rat-FAAH; PDB code: 3OJ8), from which we built a hFAAH model with MOE. In addition, the use of MODELLER allowed to interrogate several templates (i.e., PDB codes 2WJ1, 1MT5, 1M21, 1OCK), in order to increase model accuracy [30,54,55,56,57,58]. Indeed, by means of the latter software five models of hFAAH were built and analyzed with the scoring function Discrete Optimized Protein Energy (DOPE), that is largely used to check disallow regions in homology models [59]. Then, we used Procheck (stereochemical quality), Verify3D (1D-3D compatibility of an atomic model) and Errat (non-bonded interactions quality) software, in order to evaluate the quality of three selected structures: the one obtained via MOE, and two obtained via MODELLER and showing the best DOPE score (i.e., Model1-68268.296875, and Model4-68550.140625), as reported in Table S1 [60,61,62,63].

**Figure 4:**
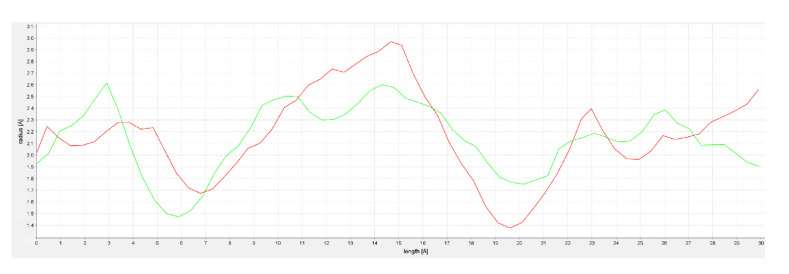
Comparison of MA channels of rat (red) and human (green) FAAHs.

In this context, it should be noted that torsion angles are among the most important local structural parameters that drive protein folding [64]. Ramachandran plot analysis of such torsion angles showed that the two MODELLER structures had a higher percentage of residues (90.8% and 91.8%, respectively) in favorable regions [65]. Furthermore, evaluation of the selected models of hFAAH was performed also through docking of the known inhibitor QK5 [48,66], leading to Model1 as the best candidate to dock cannabis-derived compounds. Overall, the analysis of the latter hFAAH model suggested that the main MA channel was tighter and longer than that of rFAAH (Figure 4), when using CAVER Analyst 2.0 to obtain channel profiles [67].

### 2.3. Molecular Docking of hFAAH

**Figure 5:**
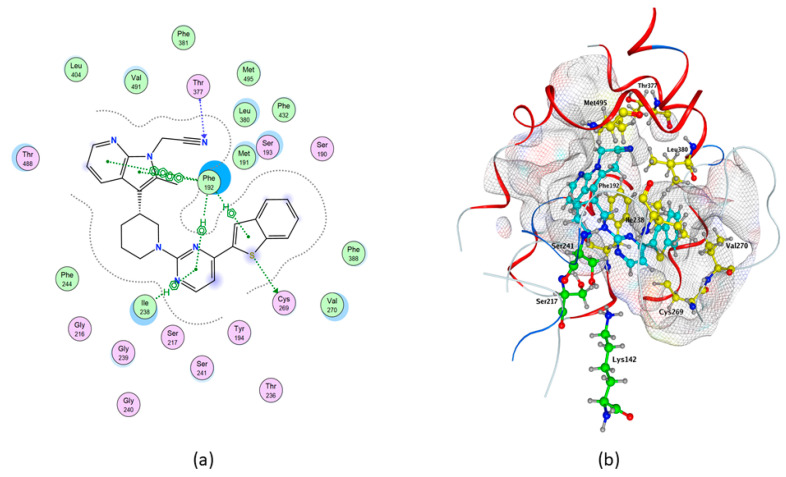
Best binding pose of QK5 within hFAAH Model1. (**a**) In the 2D structure the following details are shown: pi–H and pi–pi interactions, in green; backbone acceptor, in blue; solvent contact, in yellow. (**b**) In the 3D structure the following elements are reported as sticks: QK5, in cyan; catalytic triad, in green; all residues involved in the protein/ligand interaction, in yellow.

Molecular docking of cannabis-derived compounds was run in the active site of Model1 of hFAAH, and results are summarized in Table 3. In particular, much alike rFAAH QK5 showed the highest binding affinity to hFAAH (ΔG = −10.3235 kcal/mol), one acceptor H-bond (Thr377), one donor H-bond (Cys269), four pi–H (2 with Phe192 and 2 with Ile238) and two aromatic-aromatic (pi–pi) interactions with Phe192 (Table 2 and Table 3, and Figure 5). This compound also showed hydrophobic contacts with Ser241, Val270, Leu380 and Met495 of hFAAH (Table 2, and Figure 5). For a comparison with rFAAH, see Figure 2.

Among the cannabis-derived compounds, much alike rFAAH CBG had the highest binding affinity to hFAAH (ΔG = −8.8566 kcal/mol) without polar interactions (Table 3), and made non-polar interactions with Met191, Ile238, Phe244, Cys269, Val270, Leu278 and Val491 (Table 2). CBC showed ΔG = −8.8508 kcal/mol, one pi–H interaction with Ile238 (Table 2 and Table 3), and non-polar contacts with Leu154, Met191, Phe192, Cys269, Val270 and Leu278 (Table 2). CBN showed ΔG = −8.7723 kcal/mol, one H-donor with Ser241, two pi–H with Phe192 and two with Ile238 (Table 3), and non-polar interactions with Cys144, Met191, Phe244, Leu266, Cys269, Val270 and Leu278 (Table 2 and Table 3). CBD showed ΔG = −8.3664 kcal/mol, and non-polar contacts with Cys144, Met191, Ile238, Cys269, Val270, Leu278 and Val491 (Table 2). THCV showed ΔG = −7.8611 kcal/mol, three pi–H interactions with Phe192, 2 with Ile238 (Table 2 and Table 3), and hydrophobic interactions with Cys144, Met191, Cys269, Val270, Leu278 and Val491 (Table 2). Finally, BCP showed ΔG = −6.0123 kcal/mol (Table 3), and non-polar contacts with Leu154, Met191, Phe192, Ile238, Cys269, Val270, Leu278 and Phe388 (Table 2).

### 2.4. Activity Assays

All cannabis-derived compounds were tested at concentrations up to 100 µM on rat and human FAAH, to ascertain their inhibition potency through dose-response curves and IC_50_ values calculated thereof (Table 4). The potent and selective FAAH inhibitor URB597 (shown in Figure 1b) was used as a positive control [42].

Remarkably, CBD was found to be the most potent cannabis-derived inhibitor of rFAAH activity, with an IC_50_ value of 43.5 ± 1.5 µM that was in keeping with a previous report [46]. CBN was second in the potency ranking, with an IC_50_ of 60.0 ± 10.0 µM, whereas CBC and CBG were weak inhibitors (IC_50_ ~100 µM), and THCV and BCP were ineffective under the same experimental conditions (IC_50_ > 100 µM). At variance with rFAAH, tested cannabis-derived compounds were found to be ineffective on hFAAH activity (IC_50_ values > 100 µM), or to weakly inhibit it as in the case of CBN (IC_50_ of ~100 µM) (Table 4). For CBD, these findings extend a previous report [47].

## 3. Discussion

Cannabis constituents hold potential for their biological properties, largely due to their ability to interact with different components of the ECS. In particular, THCV and CBG have a significant affinity (in the μM range) for different TRPV channels and TRPM8 [46,68]. CBN binds to TRPA1 and TRPM8 channels, with agonistic and antagonistic effects respectively [46]. CBC is the most potent agonist of TRPA1 channels (EC_50_ = 90 nM), activates TRPV3 and TRPV4 and inhibits TRPM8 channels [68]. CBD has high affinity (in the nM range) for GPR55 and TRPM8 [46,69], a remarkable affinity (in the low μM range) for TRPV channels and TRPA1 [46,68], and quite a good affinity (in the μM range) for CB_1_, CB_2_, 5-HT 1A receptors and peroxisome proliferator-activated receptor γ [70,71,72].

Unlike eCB-binding receptors, the possible effect of cannabis ingredients on metabolic enzymes of these lipid signals, such as FAAH, have been poorly investigated. Of note, synthetic inhibitors have been shown to be much more potent on rFAAH than on hFAAH, possibly due to differences in active site and MA channel in the two enzymes [41,42,45].

Here, we first performed in silico docking analysis to evaluate the affinity of selected cannabis-derived compounds for rFAAH and an unprecedented model of hFAAH, and assessed their interactions with multiple binding cavities. Then, we assessed the ability of the same natural compounds to inhibit enzyme activity through in vitro assays. To correlate computational analysis with in vitro data, it is necessary to evaluate both binding affinity and number and types of interaction of each compound with the target protein.

**Figure 6:**
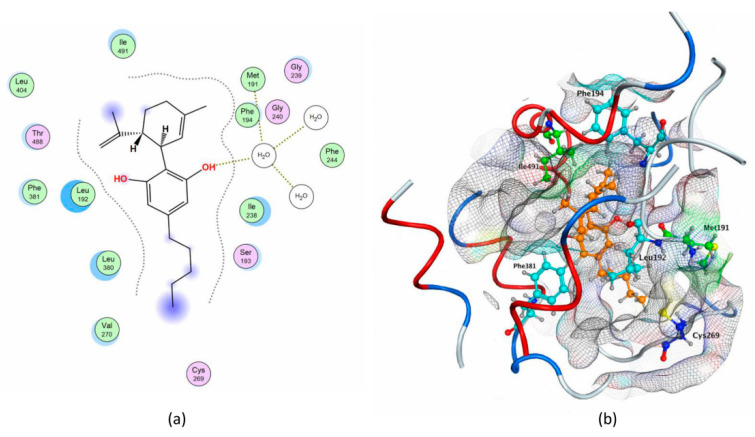
Pattern of CBD interactions with key rFAAH residues. (**a**) In the 2D structure the solvent contact is reported in yellow. (**b**) In the 3D structure the following details are reported as sticks: CBD, in orange; Leu192, Phe194 and Phe381, in cyan; Met191 and Ile491, in green; Cys269, in blue. The pictures were generated by means of the MOE software.

To this aim, we first analyzed interactions with rFAAH of QK5, a well-known potent inhibitor engaged in a series of key interactions with different binding cavities; then, we interrogated the possibility that cannabis-derived compounds could make the same interactions (Table 5). Of note, binding of CBD revealed a pattern of interactions with cavities that are important for the inhibition of rFAAH activity (Table 5 and Figure 6).

Indeed, CBD binding to rFAAH could prevent AEA movement from MA to ACB, by interacting with Met191 and blocking CP. These results are in agreement with the calculated IC_50_ value of CBD (Table 4), which extends previous reports [46,47].

Interestingly, CBN was also rather effective in inhibiting in vitro rFAAH activity (Table 4), in line with its ability to interact with key residues in the MA/ACB cavity of the enzyme (Table 5). Instead, CBG and CBC showed a similarly weak inhibition potency, and they interacted with MA, MA/ACB cavities, and OH, MA/ACB cavities respectively (Table 5). Finally, THCV and BCP were ineffective on rFAAH activity (Table 4), and interacted with OH, MA/ACB cavities, and MA/ACB cavity respectively (Table 5). Though it seems rather difficult to dissect a specific role for each enzyme cavity in determining the inhibitory power of natural compounds, the present in silico and in vitro data suggest that the best inhibitors of rFAAH should make more polar contacts with the enzyme, and should be able to interact with multiple binding cavities (most notably Met191) at once (Table 5). This hypothesis is consistent with previous studies where the properties of synthetic compounds considered to be relevant for selectivity and inhibition of enzyme activity have been discussed [38].

To further characterize the therapeutic potential of cannabis-derived compounds, we investigated their interaction with an unprecedented model of hFAAH. Interestingly, none of these substances were able to inhibit hFAAH at concentrations up to 100 µM, except CBN that showed only a modest inhibition (Table 4). Yet, in silico the same compounds showed a reasonable affinity for the enzyme (Table 3).

**Figure 7:**
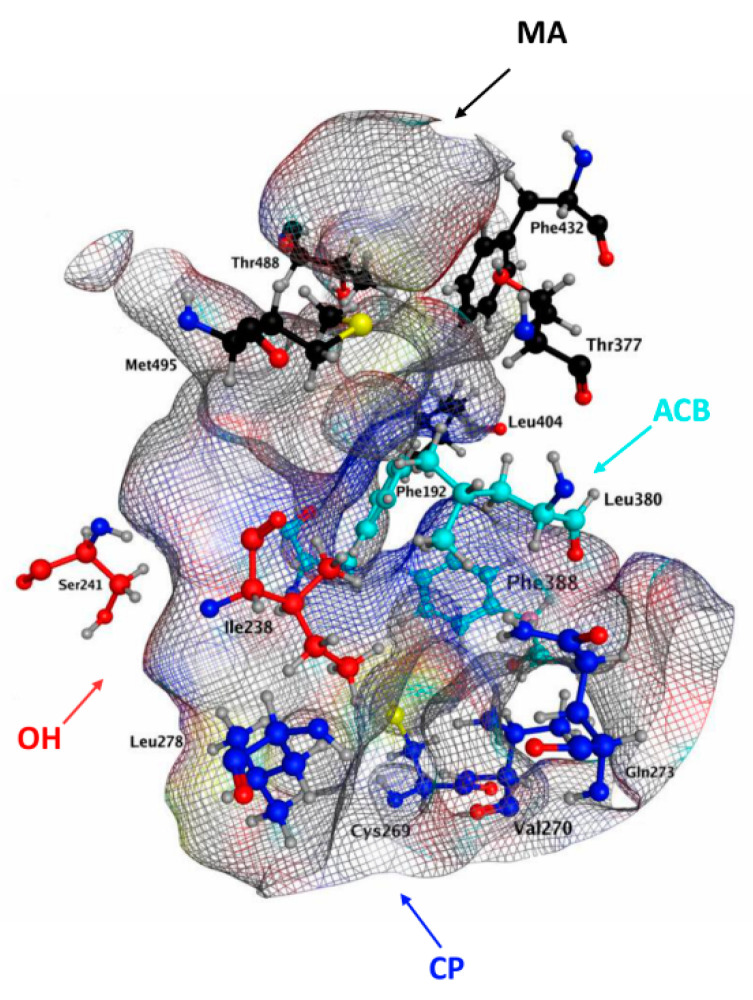
3D channels of hFAAH obtained through the MOE software. The purported key residues in the different cavities are shown as sticks. For MA, in black: Thr377, Leu404, Phe432, Thr488 and Met 495. For ACB, in cyan: Phe192, Leu380 and Phe388. For OH, in red: Ile238 and Ser241. For CP, in blue: Cys269, Val270, Gln273 and Leu278.

Unfortunately, the lack of a known 3D structure did not allow identification of the key residues of hFAAH responsible for the contacts with each cannabis-derived compound; thus, we focused on the interactions with QK5, which are well-characterized for rFAAH. By analogy with the rat enzyme, we may suggest that key residues of hFAAH could be in MA (Leu404, Thr377, Phe432, Met495 and Thr488), MA/ACB (Leu380, Phe388 and Phe192), near CP (Cys269, Val270, Gln273 and Leu278) and OH (Ile238-Ser241), as shown in Figure 7.

This hypothesis is in keeping with recent data, suggesting as key amino acids for hFAAH inhibition Phe192, Ile238, Thr377, Leu380, Phe381, Phe388 and Leu404 [47,73]. In particular, Phe at position 192 seems to be critical in the MA/ACB area, to make pi–pi and pi–H interactions with its phenyl moiety, thus driving inhibition power (Table 1 and Table 3). Instead Met191, which appears relevant for rFAAH inhibition by QK5 and CBD (Table 5), appears less (if at all) relevant to inhibit hFAAH, and indeed it is absent in the QK5 pattern of interactions with this enzyme (Figure 7). Further studies are warranted to better understand the inhibition mechanism of hFAAH, and more information on the 3D structure of this enzyme will certainly help [23].

In conclusion, through in silico and in vitro analyses we interrogated the possible structural differences and key residues involved in the interaction of rat and human FAAHs with cannabis-derived compounds. Altogether, the present results shed new light on the details involved in the different species-specific sensitivity of FAAH to potential inhibitors. They also call for caution when pre-clinical studies on rodent (rat, mouse) enzymes are translated to humans for therapeutic applications, and suggest that in silico screening of candidate FAAH inhibitors may not always deliver the best blockers of the actual in vitro enzyme activity.

## 4. Materials and Methods

### 4.1. Materials

Chemicals were of the purest analytical grade. Anandamide (AEA), URB597 and hFAAH were purchased from Cayman (Cayman Chemical Company, Ann Arbor, MI, USA). Phytocannabinoids and β-caryophyllene were purchased from Sigma Chemical Co. (St. Louis, MO, USA). [Ethanolamine-14-C]AEA was purchased from American Radiolabeled Chemicals (ARC, St. Louis, MO, USA).

### 4.2. Protein Preparation and Docking Analysis

The crystal structure of rFAAH, with a potent non-covalent co-crystallized inhibitor QK5 (PDB code: 3QK5), was retrieved from the PDB (www.rcsb.org), analyzing the Experimental Data Snapshot and the PDB validation: Resolution (2.20 Å), RFree (0.224), Clashscore (5), R-values (0.224), Ramachandran outliers (0), sidechain outliers (6.8%) and RSRZ outliers (1.5%) [48,49].

Molecular Operating Environment (MOE 2019.0102) was used to conduct the simulation studies [50]. Other molecules, as glycerol and 1,2-ethanediol, were removed from the loaded protein, whereas water molecules were kept. The selected crystal structure was prepared using the “Structure Preparation” panel, which contains the “Protonate 3D” function to optimize the ionization states of the added hydrogen atoms.

The 3D structures of the analyzed molecules were downloaded from the PubChem database (pubchem.ncbi.nlm.nih.gov) [49]. The database codes of CBG, CBD, CBN, CBC, THCV and BCP are: 5315659, 644019, 2543, 30219, 34180 and 5281515, respectively.

The molecular docking algorithm was validated by re-docking the co-crystallized ligand (QK5) in the active site. It was considered validated when the ligand conformation with the lowest energy score was superimposed to the co-crystallized molecule in the protein X-ray structure, with a RMSD < 2.0 Å [74,75].

The chosen settings in the dock panel were the Triangle Matcher method as placement, from which 30 poses were retained, and the London dG as scoring function. London dG estimates the free energy of binding of the ligand from a given pose, summing the rotational and translational entropy (*c*), the energy due to the loss of flexibility of the ligand (*E_flex_*), the geometric imperfections of hydrogen bond (*f_HB_*), the geometric imperfections of metal ligations (*f_M_*) and the desolvation energy (*D_i_*). The functional form is a sum of therms:

$$\Delta G=c+E_{flex}+\underset{h-bonds}{\sum }c_{HB}+f_{HB}+\underset{m-lig}{\sum }c_{M}f_{M}+\underset{atoms\;i}{\sum }\Delta D_{i}$$

Afterwards, an induced-fit refinement was performed, allowing both the ligand and the active site to move freely, and the poses were rescored using the GBVI/WSA dG scoring function. This forcefield-based scoring function estimates the free energy of binding using the MMFF94x and AMBER99 on the 99 protein-ligand complexes of the solvated interaction energy training set. The equation is:

$$\Delta G\approx c+\alpha \left[\frac{2}{3}\left(\Delta E_{Coul}+\Delta E_{solv}\right)+\Delta E_{vdW}+\beta \Delta SA_{weighted}\right]$$

Finally, docking scores of the best poses were recorded. The same validated procedure was used both for rFAAH and hFAAH.

### 4.3. Homology Modeling

We used homology modeling technique to construct the tridimensional model of human FAAH, since its crystal structure is not yet available. The sequence was obtained from the UniProt database (UniProt accession number O00519) [76]. Models were built using different comparative modeling approaches: fully automated web-servers (SWISS-MODEL and Phyre^2^) and two non-automated software (MODELLER and MOE) [50,51,52,53]. From several crystal structures available in PDB as template, we selected 1M21 (Peptide amidase PAM, 29.21%), 1MT5 (rFAAH, 84.73%), 1OCK (malonamidase E2, 27.31%) and 2WJ1 (hrFAAH, 85.27%) for MODELER, meanwhile MOE template is just 3OJ8 (hrFAAH, 84.53%) [30,54,55,56,57].

The quality of the generated models was evaluated using the PROCHECK, VERIFY3D, ERRAT and PROVE programs [60,61,62,63].

### 4.4. FAAH Activity Assay

Rat brains were homogenized in 50 mM Tris-HCl (50 mM, pH 7.4), and were centrifuged sequentially at 800× *g* and 20,000× *g*; then, the supernatants were discharged, and rFAAH activity was tested in membrane preparations incubated with 10 µM [^14^C]AEA at 37 °C for 15 min (pH = 9.0). The reaction was stopped with a 1:1 (*v*/*v*) mixture of chloroform/methanol and the release of [^14^C]ethanolamine in the aqueous phase was measured as reported [77]. Purified human FAAH was assayed in the same way as rFAAH. The half-maximal inhibitory concentration (IC_50_) values of each compound towards rat or human FAAH activity were calculated through non-linear regression analysis of dose-response curves (in the 0–100 μM range), performed with the Prism4^®^ program (GraphPAD Software for Science, San Diego, CA). The effect of cannabis-derived compounds on FAAH activity was ascertained by adding each substance directly to the incubation medium. Control experiments were carried out in the presence of the selective FAAH inhibitor URB597, used at 0.1 µM (for rFAAH) or 10 µM (for hFAAH), as reported [42].

## Figures and Tables

**Table 1:** Docking results of QK5 and cannabinoid-derived compounds on rFAAH.

Compounds	S (kcal/mol)	Polar Interaction	Residues	Atom Compound	Atom Receptor	Distance (Å)	E (kcal/mol)
**QK5**	−10.3374	H-acceptor	H_2_O	N	O	3.22	−1.1
		pi–H	Leu192	6-ring	C	4.31	−0.8
		pi–H	Leu404	6-ring	C	3.86	−0.9
		pi–H	Trp531	5-ring	C	3.66	−0.6
**CBG**	−8.9776	-	-	-	-	-	-
**CBD**	−8.2035	H-donor	H_2_O/Met191	O	O	3.21	−0.9
**CBC**	−7.9880	pi–H	Leu380	6-ring	C	3.78	−0.5
**CBN**	−7.7719	H-donor	Met436	O	S	3.29	−2.6
		pi–H	Phe381	6-ring	C	4.59	−0.7
**THCV**	−7.6375	-	-	-	-	-	-
**BCP**	−6.4016	-	-	-	-	-	-

S **=** score ∆G calculated Pi interactions **=** non-covalent interaction involves π systems.

**Table 2:** Interaction summary of key rFAAH residues in both enzymes with QK5 and cannabis-derived compounds.

Cavities	Residues r/h	QK5		CBG		CBD		CBC		CBN		THCV		BCP	
		rFAAH	hFAAH	rFAAH	hFAAH	rFAAH	hFAAH	rFAAH	hFAAH	rFAAH	hFAAH	rFAAH	hFAAH	rFAAH	hFAAH
**MA**	**Asp403**	-	-	-	-	-	-	-	-	-	-	-	-	-	-
	**Arg486**	-	-	-	-	-	-	-	-	-	-	-	-	-	-
	**Ile407**	✔	-	✔	-	-	-	-	-	-	-	-	-	-	-
**MA/ACB**	**Leu/Phe192**	✔	✔	-	-	✔	-	✔	✔	✔	✔	✔	✔	✔	✔
	**Phe/Tyr194**	✔	-	-	-	✔	-	-	-	-	-	-	-	✔	-
	**Phe381**	✔	✔	-	-	✔	-	✔	-	✔	-	✔	-	-	-
	**Phe432**	-	-	-	-	-	-	✔	-	-	-	✔	-	-	-
	**Met436**	✔	-	✔	-	-	-	-	-	✔	-	✔	-	-	-
	**Trp 531**	✔	-	✔	-	-	-	-	-	✔	-	-	-	-	-
**OH**	**Ile 238**	✔	✔	-	✔	-	✔	✔	✔	-	✔	-	✔	✔	✔
	**Gly239**	-	-	-	-	-	-	-	-	-	-	-	-	-	-
	**Gly240**	-	-	-	-	-	-	-	-	-	-	-	-	-	-
	**Ser241**	-	✔	-	-	-	-	-	-	-	✔	-	-	-	-
**OIR**	**Met191**	✔	-	-	✔	✔	✔	-	✔	-	✔	-	✔	-	✔
	**Ile/Val491**	✔	✔	-	✔	✔	✔	✔	-	✔	-	✔	✔	✔	-
	**Val/Met495**	-	✔	-	-	-	-	-	-	✔	-	-	-	-	-
	**Ser218**	-	-	-	-	-	-	-	-	-	-	-	-	-	-
**CP**	**Thr236**	-	-	-	-	-	-	-	-	-	-	-	-	-	-
	**Cys269**	-	✔	-	✔	✔	✔	-	✔	-	✔	-	✔	-	✔

MA: membrane access; ACB: acyl-chain binding; OH: oxyanion hole; CP: cytosolic port; OIR: other important residues.

**Table 3:** Docking results of QK5 and cannabinoid-derived compounds on hFAAH.

Compounds	S (kcal/mol)	Interaction	Residues	Atom Compound	Atom Receptor	Distance (Å)	E (kcal/mol)
**QK5**	−10.3235	H-donor	Cys 269	S	S	3.46	−1.1
		H-acceptor	Thr 377	N	C	3.73	−0.5
		pi–H	Phe 192	6-ring	C	4.28	−0.6
			Phe 192	5-ring	C	4.2	−1.1
			Ile238	6-ring	N	4.37	−1.7
			Ile238		C	3.7	−0.7
		pi–pi	Phe 192	5-ring	6-ring	3.86	0
			Phe 192	6-ring	6-ring	3.97	0
**CBG**	−8.8566	-	-	-	-	-	-
**CBC**	−8.8508	pi–H	Ile238	6-ring	C	4.23	−0.7
**CBN**	−8.7723	H-donor	Ser241	O	O	3.22	−0.5
		H-pi	Phe192	C	6-ring	4.23	−0.5
		pi–H	Phe192	6-ring	C	4.26	−1
			Ile238		C	3.53	−0.5
			Ile238		N	4.33	−0.8
**CBD**	−8.3664	-	-	-	-	-	-
**THCV**	−7.8611	pi–H	Phe192	6-ring	C	4.35	−0.7
			Ile238		N	4.21	−0.5
			Ile238		C	3.49	−0.5
**BCP**	−6.0123	-	-	-	-	-	-

S = score ∆G calculated Pi interactions= non-covalent interaction involves π systems.

**Table 4:** Inhibition of rFAAH and hFAAH by cannabis-derived compounds.

Compound	IC_50_ (µM) Towards rFAAH	IC_50_ (µM) Towards hFAAH
**CBD**	43.5 ± 1.5	>100
**CBN**	60.0 ± 10.0	~100
**CBC**	~100	>100
**CBG**	~100	>100
**THCV**	>100	>100
**BCP**	>100	>100

N.B. URB597, used as a positive control, inhibited both rFAAH and hFAAH activity > 99.5% compared to controls.

**Table 5:** Summary of in silico and in in vitro results on the interaction of tested cannabis-derived compounds with rFAAH.

	S (Kcal/Mol)	Total Key Interactions	Polar Key Interactions	% In Vitro Inhibition (At 100 µm)	Binding Cavities
**QK5**	−10.3374	9	3	N.A.	MA, OH, MA/ACB, Met191
**CBD**	−8.2035	6	1	94	MA/ACB, CP, Met191
**CBN**	−7.7719	6	2	65	MA/ACB
**CBG**	−8.9776	3	0	53	MA, MA/ACB
**CBC**	−7.988	5	0	51	OH, MA/ACB
**THCV**	−7.6375	5	0	30	MA/ACB
**BCP**	−6.4016	4	0	18	OH, MA/ACB

S = score ∆G calculated. N.A., not applicable.

## Data Availability

Please refer to suggested Data Availability Statements in section “MDPI Research Data Policies” at https://www.mdpi.com/ethics.

## Supplementary Materials

The following are available online, Figure S1: Overlay of MOE best pose (yellow) with the co-crystal structure of QK5 (green). The catalytic triad is shown as sticks: Lys142 is in red, Ser217 is in magenta and Ser241 is in cyan., Table S1: Validation of comparative models.
