# Supplementary material for: In Silico and In Vitro Analysis of Major Cannabis-Derived Compounds as Fatty Acid Amide Hydrolase Inhibitors
Source: Molecules. 2020 Dec 24;26(1):48. doi: 10.3390/molecules26010048 (PMC7795171; doi:10.3390/molecules26010048)
Supplement: Supplementary file 1 [file molecules-26-00048-s001.pdf]

Supplementary Materials

Figure S1: Overlay of MOE best pose (yellow) with the co-crystal structure of QK5 (green). The catalytic triad is shown as sticks: Lys142 is in red, Ser217 is in magenta and Ser241 is in cyan.

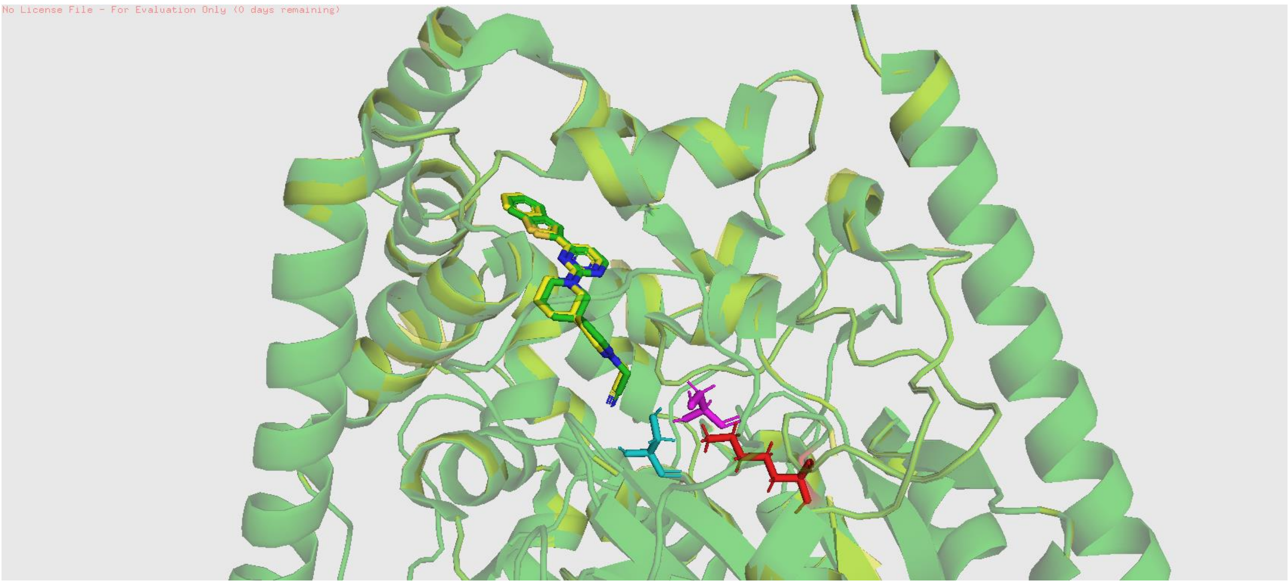

Table S1

Validation of comparative models

		**Ramachandran Plot**		**Errat** (Overall Quality Factor)	**Verify3D** (3D-1D score >0.2%)	**PROVE** (buried outlier protein atoms total from Model1)
		Residues in most favored region (%)	Residues in disallow region (%)			
SWISS-MODEL		90.3	0.2	93.3333	91.72	3.7
PHYRE2		89.3	0.4	87.7402	94.99	5.5
MOE		86.2	0.0	91.7603	92.62	3.0
MODELLER	Model1	90.8	0.4	93..5185	85.15	5.0
	Model4	9108	0.2	94.8529	87.39	4.0
